# Electric pulse characteristics can enable species recognition in African weakly electric fish species

**DOI:** 10.1038/s41598-018-29132-z

**Published:** 2018-07-17

**Authors:** Rebecca Nagel, Frank Kirschbaum, Volker Hofmann, Jacob Engelmann, Ralph Tiedemann

**Affiliations:** 1 Unit of Evolutionary Biology and Systematic Zoology, Institute of Biochemistry/Biology, University of Potsdam, 14476 Potsdam, Germany; 2 Faculty of Life Sciences, Albrecht Daniel Thaer-Institute of Agricultural and Horticultural Sciences, Unit of Biology and Ecology of Fishes, Humboldt University of Berlin, 10115 Berlin, Germany; 3 Active Sensing, Faculty of Biology, Cognitive Interaction Technology – Center of Excellence, Bielefeld University, 33602 Bielefeld, Germany; 4 Present Address: Faculty of Medicine, Department of Physiology, McGill University, H3G1Y6 Montreal, Quebec, Canada

## Abstract

Communication is key to a wide variety of animal behaviours and multiple modalities are often involved in this exchange of information from sender to receiver. The communication of African weakly electric fish, however, is thought to be predominantly unimodal and is mediated by their electric sense, in which species-specific electric organ discharges (EODs) are generated in a context-dependent and thus variable sequence of pulse intervals (SPI). While the primary function of the electric sense is considered to be electrolocation, both of its components likely carry information regarding identity of the sender. However, a clear understanding of their contribution to species recognition is incomplete. We therefore analysed these two electrocommunication components (EOD waveform and SPI statistics) in two sympatric mormyrid *Campylomormyrus* species. In a set of five playback conditions, we further investigated which components may drive interspecific recognition and discrimination. While we found that both electrocommunication components are species-specific, the cues necessary for species recognition differ between the two species studied. While the EOD waveform and SPI were both necessary and sufficient for species recognition in *C. compressirostris* males, *C. tamandua* males apparently utilize other, non-electric modalities. Mapped onto a recent phylogeny, our results suggest that discrimination by electric cues alone may be an apomorphic trait evolved during a recent radiation in this taxon.

## Introduction

Communication signals that convey sex and species identity from a sender to a receiver are vital to intra- and interspecific recognition and discrimination, often driving reproductive and behavioural isolation^1–3^. Understanding the communication mechanisms responsible for species recognition is therefore of importance for our understanding of both speciation and hybridization events^4^.

African weakly electric fish communicate by means of a pulse-like electric organ discharge (EOD) generated by a specialized electric organ situated in the caudal peduncle of adult fish^5,6^. This form of communication, termed *electrocommunication*, is of particular interest for communication studies because these electric signals can be reliably recorded under natural conditions, as well as be reproduced and manipulated in experimental settings.

Two major components of mormyrid electrocommunication are thought to carry information – the EOD and the temporal pattern in which EODs are emitted, i.e. the sequence of inter-pulse-intervals (sequence pulse interval, SPI). The EOD waveform is species-specific, and the duration, number, and polarity of phases in the discharge are determined by the genetic (e.g., expression pattern of ion channels)^7^ and morphological (e.g., histology of electrocytes)^8,9^ structure of the electric organ. Electrolocation is generally considered the primary function of the electric discharges^6^. Given the intraspecies stability but notable interspecies variation in the EOD waveform, numerous studies have suggested its additional importance in mate selection and species recognition^10–13^. In contrast, the SPI is a volitional component and varies within an individual depending on behavioural context (reviewed in ref.^14,15^). Changes in the SPI in mormyrid fish have been correlated with, for example, different swimming patterns and feeding behaviours^16–18^, in response to external electrical stimulation^19^, aggressive and agonistic interactions^20–22^, courtship displays^23,24^, and solving sensory tasks^25,26^. A wide range of SPI patterns observed at short timescales (e.g., bursts, cessations, and/or regularizations of approximately 20 inter-pulse-intervals) have also been defined and linked to specific behavioural contexts^14,27^.

Notwithstanding this large body of research addressing numerous aspects of mormyrid electrocommunication, a clear understanding of the contribution of the two major electrocommunication components (EOD waveform and SPI) for species recognition has not yet been achieved (but see ref.^23^ for experiments with the sound producing mormyrid *Pollimyrus isidori*). In this study, we therefore investigate the contributions of these components in two African weakly electric fish species, *Campylomormyrus compressirostris* and *Campylomormyrus tamandua*.

**Figure 1:**
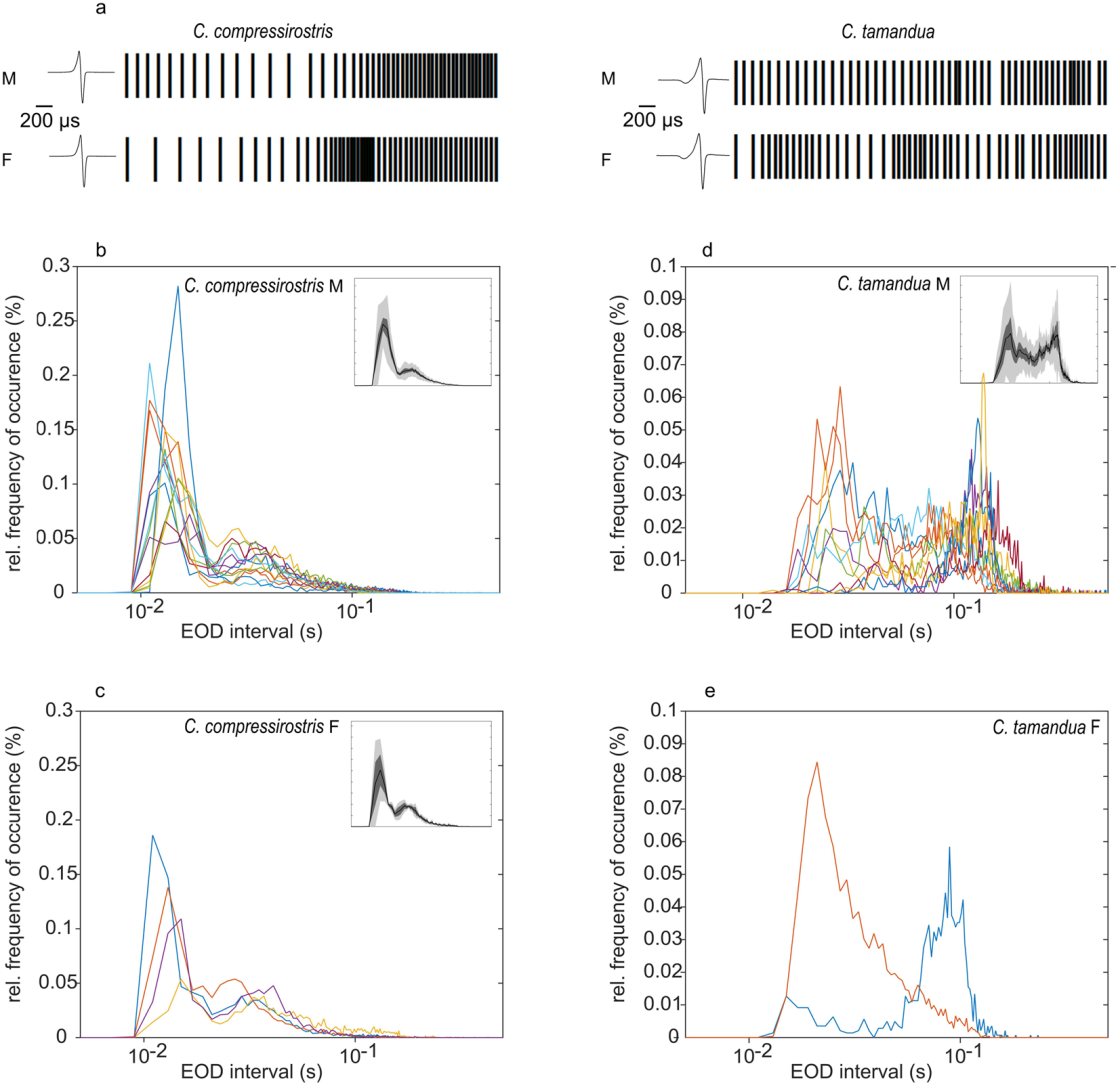
Inter-pulse-interval (IPI) histograms from resting phase SPIs show species-specific differences. Panels show relative histograms (bin width 2 milliseconds) of the IPI for a given species and sex. (**a**) A 1 second excerpt of a recorded SPI from each species and sex, where each vertical bar represents an emitted EOD. The EOD waveform visualized represents the characteristic waveform of each species. Note the initial head-negative pre-phase of low amplitude in the *C. tamandua* EOD waveform. (**b**–**e**) The IPI histograms from resting phase SPIs. Each coloured line represents a single individual. Plot insets show the population averages (with SEM: dark grey; STD: light grey). (**b**) *C. compressirostris* males (n = 13) (**c**) *C. compressirostris* female (n = 4) (**d**) *C. tamandua* male (n = 10) and (**e**) *C. tamandua* female (n = 2; population averages not shown because of small sample size). F = female; M = male.

*Campylomormyrus* species are endemic to the river systems of the Congo, Niger, Chad, Shari, Zaïre, and Volta, and both *C. compressirostris* and *C. tamandua* occur in sympatry in the Congo Basin^28,29^. While most morphological divergence between these two species is confined to the snout, the EOD waveforms differ significantly^28,30^. The biphasic *C. compressirostris* EOD waveform is characterized by a short, head-positive peak followed by a second head-negative phase with an average duration of 164 μs. The EOD waveform of *C. tamandua*, in contrast, is triphasic with an initial head-negative pre-phase of low amplitude followed by a head-positive phase and a final head-negative phase. The EOD has an average total duration of 316 µs (Fig. 1a). Neither species displays a sexually dimorphic EOD waveform^8^.

Previous research on *C. compressirostris* and *C. tamandua* suggests that males may mediate species recognition and pre-zygotic reproductive isolation^31^. It is, however, as of yet unknown which components of the electrocommunication signal are necessary for discrimination or how the EOD and SPI components interact for successful species recognition. In the current study, we therefore investigated SPI statistics recorded from *C. compressirostris* and *C. tamandua* individuals. In contrast to the stereotypic SPI patterns that have been successfully defined at short timescales^14,27^, our aim was to investigate SPI statistics at longer timescales and determine if (1) these statistics differ between species and if so, (2) if species can recognize and distinguish between these differences. In playback experiments, we further tested the behavioural relevance and contribution of each of the electrocommunication components (EOD waveform *vs* SPI) for species recognition. Our findings establish that not only the EOD waveform but also the SPI statistics differ significantly between these two species, but that those components responsible for species recognition and discrimination vary depending on species.

## Results

### SPI statistics

To establish possible species and sex differences in SPI statistics during electrocommunication, 29 SPI trains were recorded from unrestrained, isolated *C. compressirostris* (male n = 13, female n = 4) and *C. tamandua* (male n = 10, female = 2) individuals during the resting phase, i.e. during the day. Six additional SPI trains were recorded from unrestrained, isolated female *C. compressirostris* (n = 4) and female *C. tamandua* (n = 2) during the active phase, i.e. at night. A segment (time span: 1 second) of a resting phase SPI for each species and sex, where each vertical line represents a generated EOD, is shown in Fig. 1a. From two individuals, three 30-minute SPI trains were recorded on separate dates to show individual variation in SPI statistics; a summary of these results is provided in the Supplementary Information Table S1.

A visual analysis of the SPI patterns from *C. compressirostris* individuals suggests that during the resting phase, inter-pulse-intervals follow trends, such as cycles of bursts or periods of highly variable inter-pulse-intervals (IPIs; cf. formula 1 in methods). In *C. compressirostris* individuals, bursts were often followed by short periods of regularized IPI sequences. *C. tamandua* individuals recorded during the resting phase displayed a broad range of intervals and produced notably fewer bursts than *C. compressirostris*. For the SPI trains recorded during the active phase, less variation in the IPI was observed in both species (Supplementary Information Fig. S1).

From the resting phase SPI recordings, EOD interval histograms were produced for each species and sex to visualize absolute changes in IPI distributions. For both male and female *C. compressirostris* individuals, the histograms were bimodal with prominent peaks at 14 and 23 milliseconds, respectively (Fig. 1b,c). In contrast, the resting phase SPIs of *C. tamandua* individuals were broadband, with intervals occurring at a relatively similar probability between 25 and 150 milliseconds. Clear predominant modes like those seen in *C. compressirostris* were not consistently apparent across *C. tamandua* individuals, but the comparison of individual histograms suggests that an alternating activity pattern with transitions between long and short intervals might occur (Fig. 1d,e; for details see Supplementary Information Fig. S2).

**Figure 2:**
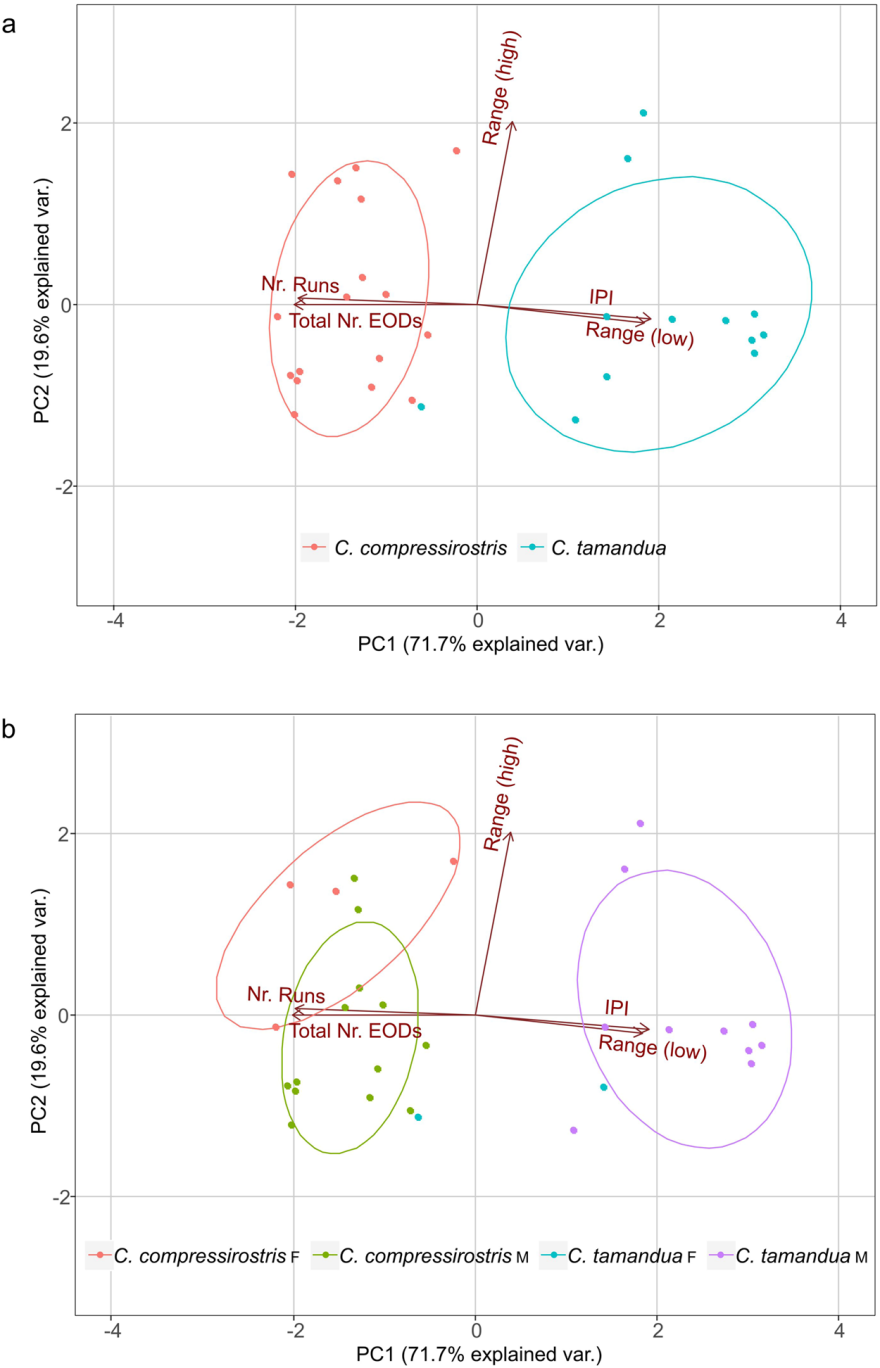
Principle components of SPIs differ between species and sex. Five variables (see Table 1) were examined across the 29 resting phase SPIs recorded from *C. compressirostris* and *C. tamandua* individuals. (**a**) A comparison between the two species: *C. compressirostris* (n = 17; red) and *C. tamandua* (n = 12; blue). (**b**) A comparison between the two species, including sex as a group variable, with *C. compressirostris* females (n = 4; red), *C. compressirostris* males (n = 13; green), *C. tamandua* females (n = 2; blue) and *C. tamandua* males (n = 10; purple). PC = principle component; F = female; M = male.

Given potential species-specific differences in the IPIs, the 29 resting phase SPIs were further analysed using a principle component analysis (PCA). Five variables were extracted from each individual SPI train: (1) the total number of EODs produced in the 120-second sequence, (2) the mode inter-pulse-interval (i.e. the most frequent EOD interval), (3 & 4) the range (i.e. longest and shortest interval between two adjacent EODs), and (5) the number of runs, as derived from the runs test (please see Table 1 and the Methods for more detailed information). First, to identify those variables that may explain variability between the two species’ SPIs, sex was ignored and the *C. compressirostris* and *C. tamandua* species were compared. The two species form distinct clusters and are divided primarily along PC1 (Fig. 2a). Accounting for 71.7% of variance between species, PC1 is loaded mainly with the total number of EODs (loading: −0.517) and the predominant inter-pulse-interval (loading: 0.492). Downstream analysis shows that *C. tamandua* individuals did produce significantly fewer EODs in the 120-second recording period than *C. compressirostris* (two sample *t*-test; *p* < 0.001). Second, to identify any sex differences in the SPIs, we performed a subsequent, within-species PCA. The male and female *C. compressirostris* SPIs show a weak but insignificant division along PC2, loaded almost exclusively with the recorded range (i.e. the largest inter-pulse-interval for a given individual) (loading: 0.992) (Fig. 2b). Given that only two SPIs could be recorded from *C. tamandua* females, a statistical comparison across sex was not possible within this species. A Pearson correlation matrix and probability values for each pair of variables is provided in the Supplementary Information Table S2.

**Table 1:** Definitions of the five variables examined for the principle component analyses of resting phase SPIs and their group mean ± SE.

Description of term	Total Nr. EODS	Nr. Runs	IPI	Range (high)	Range (low)
	total number of EODs recorded in the 120-second resting phase SPI	total number of runs in the SPI; a run was defined as a series of increasing, decreasing or constant EOD interval times (see Methods for more details)	predominant (mode) inter-pulse-interval recorded in the SPI	longest interval between two adjacent EODs	shortest interval between two adjacent EODs
*C. compressirostris* M; n = 13	3157 ± 116	1836 ± 72	23 ± 4 ms	394 ± 101 ms	10 ± 0.190 ms
*C. compressirostris* F; n = 4	3131 ± 434	1987 ± 296	14 ± 1 ms	1062 ± 271 ms	10 ± 0.005 ms
*C. tamandua* M; n = 10	1303 ± 96	855 ± 71	104 ± 10 ms	790 ± 281 ms	17 ± 0.949 ms
*C. tamandua* F; n = 2	1238 ± 717	1300 ± 370	48 ± 25 ms	194 ± 38 ms	14 ± 0.119 ms

**Figure 3:**
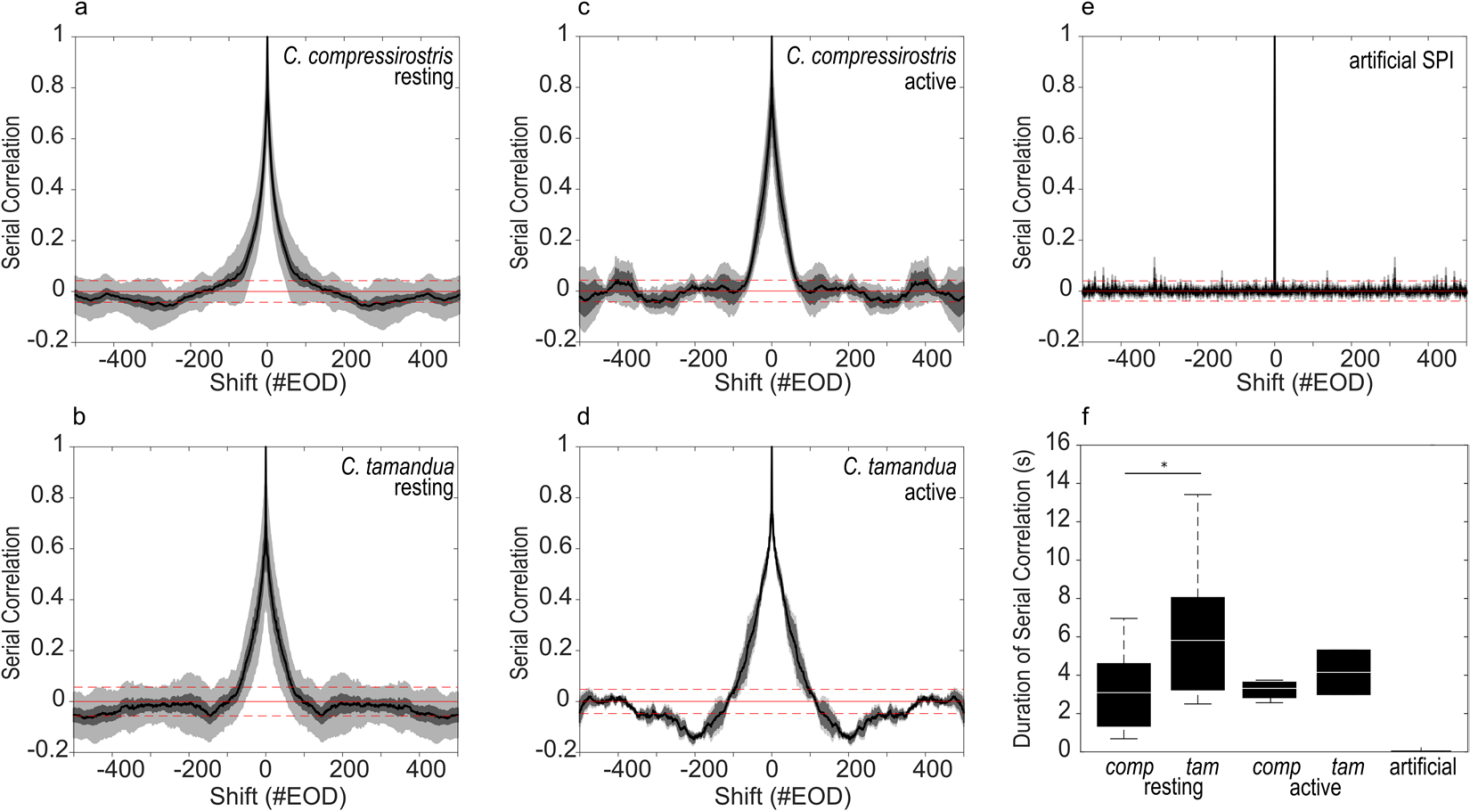
SPI serial correlation durations differ between species. (**a**–**d**) Average serial correlations for 120-second recordings of the discharge behaviour of *C. compressirostris* (a: resting, n = 17; c: active, n = 4) and *C. tamandua* (b: resting, n = 12; d: active, n = 2). Population averages are shown with SEM (dark grey) and STD (light grey). The red line depicts the chance level (at a type I error rate of *p* = 0.01) at which serial correlations could be found in the respective datasets. (**e**) Same as (**a**–**d**), but for the artificial (scrambled) SPIs (n = 6). (**f**) Distribution of average serial correlation durations. Stars (*) represent significance. *comp* = *C. compressirostris*; *tam* = *C. tamandua*.

Under the assumption that the SPI component of the electric activity contains information for a receiver, we calculated serial correlations (SC) of the SPI trains to test if the sequential order of emitted EODs is influenced by memory effects (i.e. a dependency of a given EOD interval and its past) or if EOD emission is a renewal process (i.e. EOD intervals occur randomly). For all natural (recorded and unaltered) SPIs, we found serial correlations that exceeded chance level (at a type I error rate of *p* = 0.01) and could extend up to approximately 100 inter-pulse-intervals (IPIs; Fig. 3a–d). In contrast, the artificial (scrambled) SPIs are a renewal process, with the serial correlation never increasing above or decreasing below chance level (at a type I error rate of *p* = 0.01; Fig. 3e). We also used the median IPI to calculate the duration of SC for each individual. These differed between species and were significantly smaller for *C. compressirostris* than for *C. tamandua* in resting phase SPIs (Fig. 3f; Kruskal-Wallis test; *p* = 0.008). The same trend was evident for SPIs recorded from active fish, but the difference was not statistically significant (Fig. 3f; Kruskal-Wallis test; *p* = 0.064). We suspect the limited volume of data available for active fish (n = 6) prevents a clearer distinction. The serial correlation durations within a species did not significantly differ depending on activity phase (Kruskal-Wallis test; *p* = 0.361 (*C. tamandua*) and *p* = 0.474 (*C. compressirostris*)).

### Behaviour Choice Experiments

**Figure 4:**
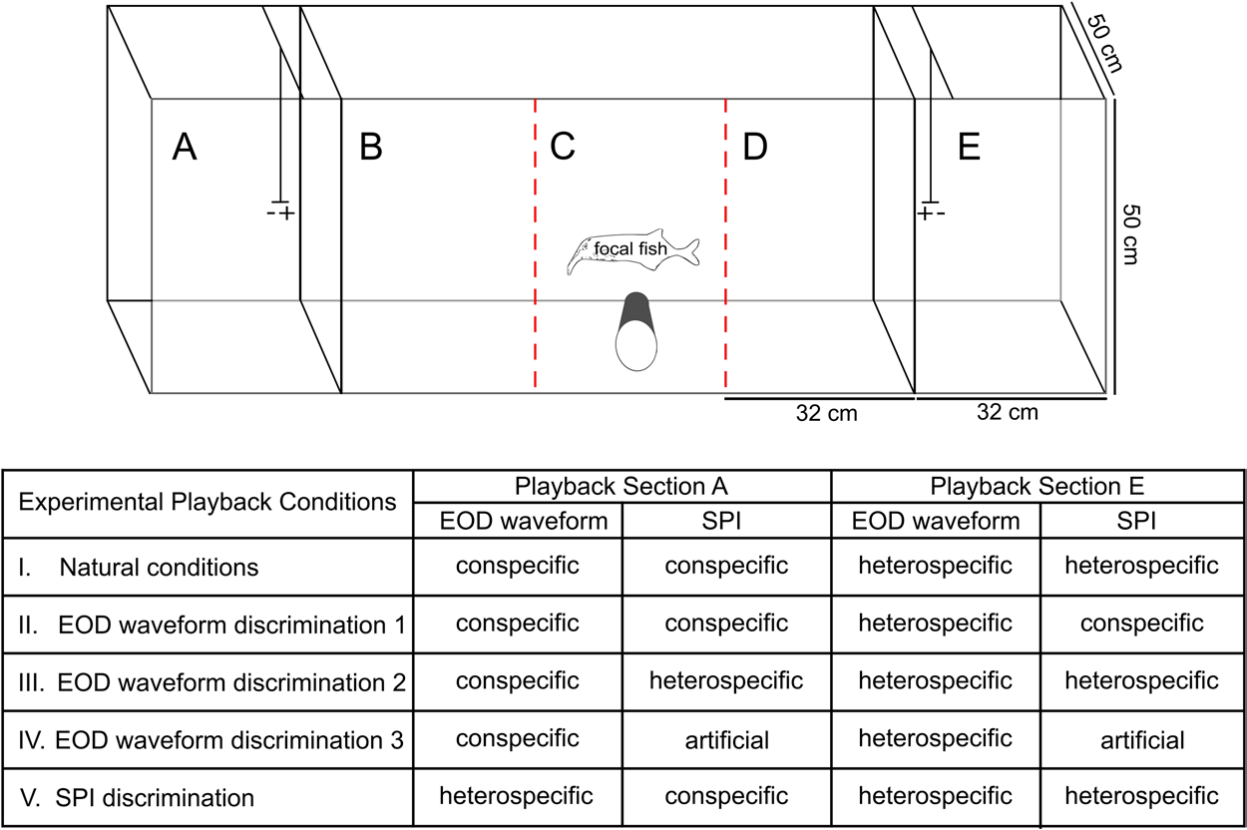
Experimental playback setup. The aquarium used for playback experiments was divided into five sections (marked A–E). The playback electrodes were located behind gridded partitions in sections A and E, with the positive pole facing the centre of the aquarium. The focal fish was positioned in the centre, which was divided in space into three equal sections (marked B–D). Sections B and D represent the respective preference zones for A and E; section C was considered a neutral zone. The five (I–V) playback conditions conducted are listed in the table, along with the EOD waveform and SPI combinations. During playback experiments, the actual physical location (section A *vs*. E) of the synthesized playback combinations was randomly interchanged to control for side-bias. EOD = electric organ discharge; SPI = sequence pulse interval.

To investigate the contribution of both electrocommunication components (EOD waveform and SPI) towards species recognition in *C. compressirostris* and *C. tamandua*, we presented sexually mature male individuals with different communication stimuli consisting of combinations of an EOD waveform (con- or heterospecific) and SPI (conspecific, heterospecific, or artificially scrambled). The two differing communication stimuli were presented simultaneously through spatially separated playback electrodes. Based on video recordings under IR illumination, the responsiveness of male fish (i.e. association time near each playback electrode) was evaluated post-hoc using strength of preference (SOP) values^32^. Each individual was tested in five different conditions (Fig. 4).

**Figure 5:**
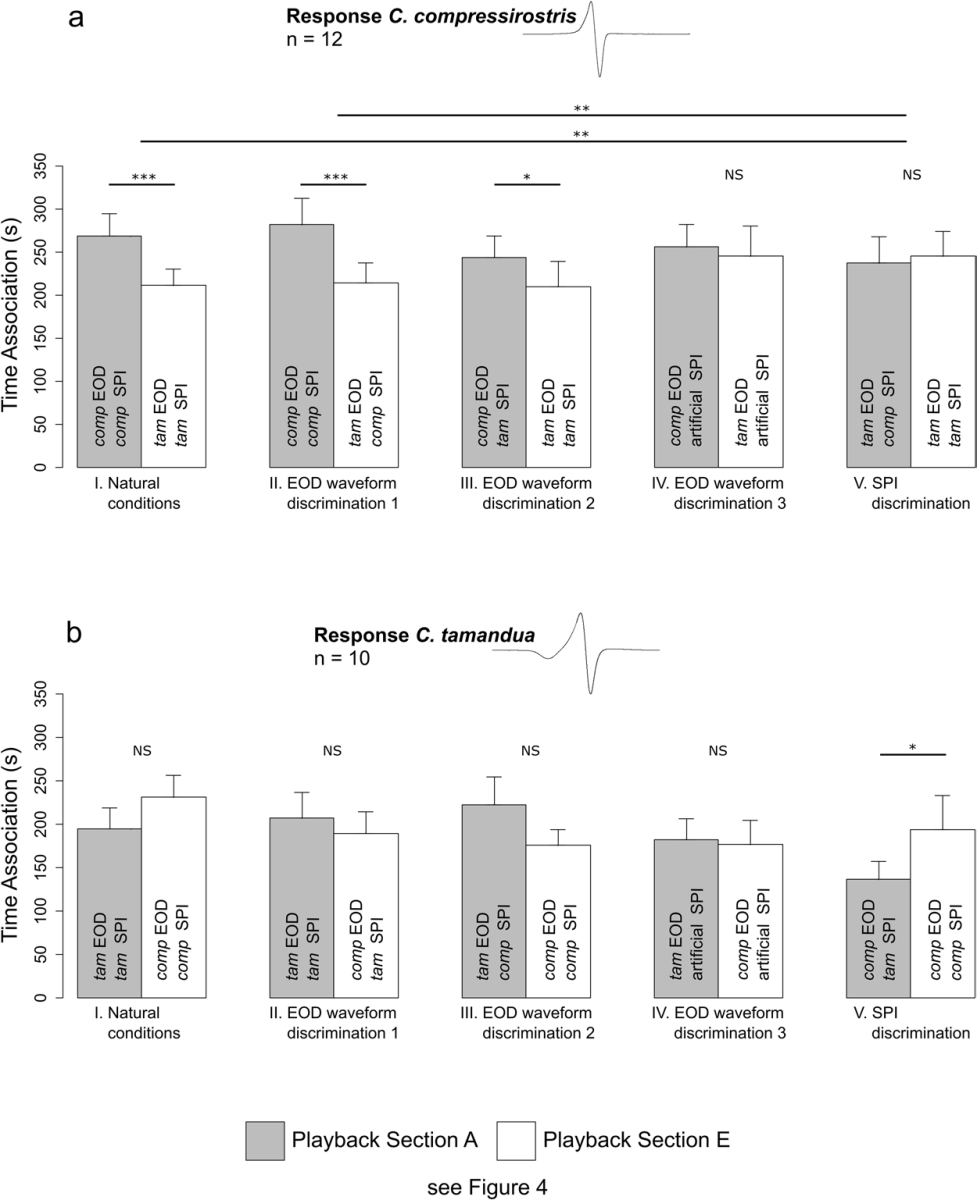
Association preferences during each of the five playback conditions. Association behaviour is displayed as mean values ± SEM. EOD insets represent the characteristic waveform of each species. (**a**) Playback experiment results for *C. compressirostris* males (n = 13). (**b**) Playback experiment results for *C. tamandua* males (n = 10). Stars (*) represent significance. EOD = electric organ discharge; SPI = sequence pulse interval; *comp* = *C. compressirostris*; *tam* = *C. tamandua*.

During playback conditions, *C. compressirostris* males (n = 12) spent significantly more time associating with the conspecific EOD waveform, regardless if the playback was combined with a con- or heterospecific SPI (Fig. 5a; one-sample *t*-test against a random distribution of SOP = 0; *p* < 0.001 in conditions I and II; *p* = 0.031 in condition III). However, if the SPI was artificial (scrambled), no significant preference between the two playback electrodes was detected (Fig. 5a; *p* = 0.159 in condition IV). The playback of heterospecific EOD waveforms combined with a conspecific SPI or a heterospecific *C. tamandua* SPI also elicited no significant association preference (Fig. 5a; *p* = 0.672 in condition V). Pairwise comparisons of the SOP values across conditions identified a significantly different response between condition I (natural conditions) and V (SPI discrimination), and condition II (EOD waveform discrimination 1) and V (SPI discrimination) (Fig. 5a; Tukey HSD; *p* = 0.009 and 0.003, respectively). In summary, *C. compressirostris* males spent significantly more time near the conspecific signal only if the EOD waveform was paired with a natural (recorded and unaltered) SPI.

During playback recordings with *C. tamandua* (n = 10), male individuals displayed no significant association preference during the first four experimental playback conditions (Fig. 5b; one-sample *t*-test against a random distribution of SOP = 0; *p* = 0.130 in condition I; *p* = 0.305 in condition II; *p* = 0.346 in condition III; *p* = 0.145 in condition IV). In condition V for SPI discrimination, *C. tamandua* individuals showed a significant association preference for the heterospecific *C. compressirostris* EOD waveform and SPI (Fig. 5b; one-sample *t*-test against a random distribution of SOP = 0; *p* = 0.049). No significant differences in SOP values across conditions was identified (ANOVA on arcsine transformed SOP values; all *p*-values > 0.100). In summary, *C. tamandua* males did not show a significant association preference for the conspecific EOD waveform or SPI.

During playback conditions, the responsiveness of *C. compressirostris* and *C. tamandua* males to the playback signals, calculated as the total respective time spent outside of the neutral zone, did not significantly differ across the five conditions (ANOVA on square transformed time data; *p* = 0.968 and 0.645, respectively).

## Discussion

Over three decades ago, Hopkins and Bass (ref.^10^) established that species recognition can be mediated by the electric organ discharge (EOD) and sequence pulse interval (SPI), the two major components of electrocommunication in mormyrid fish. Here, we present further evidence that both of these electrocommunication components exhibit species-specific features. We also suggest that while they may be relevant for species recognition and discrimination, the cues mediating recognition may vary depending on species.

While the adult EOD waveform clearly differs between species, differences in the SPI are not as easily quantifiable. SPIs are volitional and context dependent, related to the dual function of discharges in electrocommunication and electrolocation. Nonetheless, a wide range of stereotypic SPI patterns have been categorized in mormyrid species, including bursts (brief accelerations, smooth accelerations, rasps, etc.), cessations, and regularized intervals of EODs (see, e.g., Table 2 in ref.^27^). Here, we could also identify similar patterns qualitatively. Most notable was the tendency of resting *C. compressirostris* to produce bursts, while resting *C. tamandua* displayed a more heterogeneous discharge pattern. Additional analyses of the SPI across a longer timescale also suggest that certain aspects of the resting phase SPI are species-specific, such as the average EOD frequency. We were also able to quantify a species-specific difference in the average duration of SPI serial correlations in the two investigated species, which are shorter in *C. compressirostris* than *C. tamandua*. This coincides with a significantly lower number of total discharges in the 120-second *C. tamandua* recordings compared to *C. compressirostris*. This species specificity suggests that the SPI may transmit relevant information to the receiver, possibly regarding species identity. Previous studies on a wide range of mormyrid species have already suggested that the sequential manner of impulse discharges can carry information, with alternative EOD interval patterns hypothesized to correlate with sex, age, and/or dominance status^16,23,27,33–37^. Specific SPI patterns have also been linked to courtship and spawning behaviour (*Pollimyrus*)^23,24,38^ and various other social interactions (*Brienomyrus*^22^; *Gnathonemus*^16^; *Marcusenius*^39^).

Given the possibility that both the EOD and SPI components of electrocommunication may be relevant for species recognition and discrimination, we conducted a series of playback experiments where con- and heterospecific EOD waveforms and SPIs were artificially combined. Our results from these five conditions suggest that for *C. compressirostris* males, the EOD waveform may be the predominant cue used for species recognition (conditions I–III). Interestingly, a previous playback study found that female *C. compressirostris* do not discriminate between conspecific EOD waveforms and that of *C. tamandua*^12^. This sex-based asymmetric response to playback cues has been observed in other mormyrid studies as well. In the genus *Brienomyrus*, for example, preferential association with the conspecific EOD waveform was observed for male *B. brachyistius triphasic* and Gabon’s *Brienomyrus* species flock^10,40^ (now revised to the genus *Paramormyrops*)^41^. In contrast, females were relatively unresponsive to electrical playback signals^40^. This suggests that in both *C. compressirostris* and possibly other mormyrid species, males may mediate species recognition and discrimination.

While *C. compressirostris* males associated significantly more with the conspecific EOD waveform, their preference was not absolute. Such a pattern is commonly seen in preference tests (not only in mormyrids^11^, but also, e.g., in mollies^42,43^ and swordtails^44^), and may be a result of varying and overlaying motivations, e.g., mate search and exploratory behavior (‘selective responsiveness’)^11^. It further suggests that the fish were not avoiding proximity to heterospecific signals.

While male *C. compressirostris* showed a clear association preference for the conspecific EOD waveform when it was paired with a natural (recorded and unaltered) SPI, the conspecific EOD playback did not elicit a preference response in all conditions. When the conspecific EOD waveform was combined with an artificial (scrambled) SPI (condition IV), no association preference was evident. While a lack of response is not in and of itself evidence for a lack of perceptual discrimination, our SPI statistics confirm (as shown previously in, e.g.^23,27,33,34,36,37^) that the temporal pattern of EOD intervals is not random under natural conditions and contains species-specific features in the species and contexts studied here. The lack of response to the scrambled SPI may therefore suggest that natural SPIs carry valuable information, which is lacking when the SPI has a randomized temporal structure. Both the SPI and EOD waveform may therefore be relevant, necessary, and sufficient for species recognition and discrimination by *C. compressirostris* males.

In contrast to *C. compressirostris* males, *C. tamandua* males did not show an association preference for conspecific electric signals. Only in condition V, where the heterospecific EOD waveform was combined with a con- or heterospecific SPI, did males show a greater strength of preference for the heterospecific *C. compressirostris* SPI. A type-I error could possibly explain this unexpected and marginally significant result (*p* = 0.049), or these observations may – at first glance – suggest that *C. tamandua* males are willing to undermine reproductive isolation. Sympatric species often display asymmetric sexual isolation behaviours, with one species discriminating more strongly than vice versa^45,46^. However, an effort to obtain natural hybrids in breeding groups between female *C. compressirostris* and male *C. tamandua* led to several spawning events, but never fertilization^47^. This corresponds with previous research showing that *C. tamandua* males do preferentially associate with female conspecifics (over female *C. compressirostris*) when live fish are used as stimuli^31^. A recent genetic analysis also shows no evidence of gene flow between the two species, although incipient speciation is apparent^30^. Artificial reproduction between *C. compressirostris* and *C. tamandua* individuals produces fertile offspring and F1-hybrids produce viable fish^47^.

The apparent absence of a behavioural preference by *C. tamandua* males is not *per se* evidence that the species cannot discriminate between con- and heterospecific signals, but the playback results may suggest that the cues used for species recognition differ among mormyrid species. While the EOD waveform and SPI are both relevant for species recognition and discrimination in *C. compressirostris*, electric cues alone may not be sufficient for discrimination in *C. tamandua*. Molecular phylogenies of the *Campylomormyrus* genus show *C. tamandua* as the sister species of all other *Campylomormyrus* representatives^30^ and morphological investigations into the electric organ suggest that the rostral position of the stalk found in *C. tamandua* is the ancestral state^47,48^. Discrimination by electric cues alone may, therefore, be an apomorphic trait evolved during a more recent radiation (i.e., after separation from *C. tamandua*), while electric cues alone may not be sufficient for species recognition and discrimination in *C. tamandua*.

In addition to electrocommunication, various modalities may act either synergistically with the electrosensory system or as a ‘back-up’ signal^49,50^. The lack of discrimination among *C. tamandua* males during our behavioural experiments could, therefore, be due to the absence of suitable cues of other modalities. Overt behavioural interactions, such as the distinct swimming patterns observed during courtship and spawning in the mormyrid *P. isidori* and the anti-parallel displays of dominance and aggression observed in *G. petersii* (i.e. the interaction between the electric and mechanosensory sense) may have been a key factor missing during our playback setup^24,51^.

In addition to the wide scope of studies addressing electrocommunication in mormyrid weakly electric fish, the electric gymnotiform of South America have also been extensively studied. Although these two electric fish lineages arose independently from non-electrogenic teleost ancestors after the separation of South America and Africa^52,53^, both groups show striking convergence in electric organ development^5,54–57^, reproductive behaviour^58,59^, and ecology^60,61^. While the majority of gymnotiforms have constant quasi-sinusoidal EOD waveforms, a number of species have developed pulse-like EODs similar to that in mormyrids. In those species, both components of the electrocommunication signal (EOD waveform and frequency) have been shown to play a role in species and sex recognition^62–64^.

The complexity and variation of the comparatively simple electrocommunication used by weakly electric fish for species recognition emphasizes that our understanding of inter- and intraspecies communication is still largely incomplete. Weakly electric fish are therefore a particularly interesting model for communication studies because many species occur in sympatry, where effective communication is vital for species recognition and discrimination. In our study, we have been able to unravel species-specific characteristics of the SPI and present further evidence for the contributions of the EOD waveform and SPI for *Campylomormyrus* species recognition and discrimination. Future investigations are needed to elaborate upon the various other communication modalities acting synergistically to electrocommunication in African weakly electric fish.

## Methods

### Study species

Two mormyrid weakly electric fish species were investigated in this study - *Campylomormyrus compressirostris* and *C. tamandua*. All specimens were imported from Kinshasa (Democratic Republic of the Congo), where they occur in sympatry^28^. Individuals were held in the laboratory at the University of Potsdam, Germany under a 12/12 hour dark/light cycle and fed daily with Chironomidae and *Chaoborus* larvae.

### Recordings of discharge behaviour

The inter-pulse-interval (IPI) is under volitional control and consequently varies depending on behavioural context^14,23,34^. We therefore standardized the time and conditions for recording SPIs (tank: L 32 × W 50 × H 50 cm; conductivity: 700 ± 20 μS/cm; temperature: 25 ± 1 °C). During SPI recordings, a plastic tube was placed in the tank for shelter and movement was not restricted in any way. From 17 *C. compressirostris* (n = 13 males; n = 4 females) and 12 *C. tamandua* (n = 10 males; n = 2 females), we recorded SPIs over a period of 30 minutes, four hours after light onset (i.e. within the resting phase of the day-cycle) after over-night acclimation to the tank. While recording, we visually observed the locomotor activity of the fish; the fish remained in the shelter for the majority of the duration of the recording period. Under these circumstances, the electric displays connected to electrolocation are likely very limited. We additionally recorded six SPIs from female *C. compressirostris* (n = 4) and female *C. tamandua* (n = 2) one hour after night-simulation (i.e. during the active phase). For all recordings, electrode signals were amplified (MA 102 amplifier; University of Cologne, Zoological Institute, Electronics Lab for Animal Physiology) and digitized (MICRO 1401 data acquisition unit; Cambridge Electronic Design; 100 kHz sampling rate; 16 bit amplitude resolution) to be stored on a desktop computer (Spike2 software; Cambridge Electronic Design).

Under the assumption that the SPI contains relevant information, we randomly shuffled the inter-pulse-intervals (IPI) of the six, active phase SPI recordings using the programs R and MATLAB (R2015a, MathWorks Inc., Natick, MA USA) for use in playback condition IV (see below). We opted to use a randomized pattern of natural intervals (i.e. scramble the temporal order but maintain the IPI distribution) rather than create a completely artificial SPI pattern to maintain the second order SPI statistics of the original recordings.

### Recording and setup of behaviour choice experiments

To explore which electrocommunication component (s) (EOD waveform and SPI) mediate species recognition, we artificially combined con- and heterospecific SPIs and EOD waveforms from *C. compressirostris* and *C. tamandua* in five experimental playback conditions. For the purpose of this experiment, the heterospecific signal (EOD waveform or SPI) presented to *C. compressirostris* was that of a *C. tamandua* individual, and vice versa. The artificial (scrambled) SPI presented was from conspecifics. All focal fish were males with a kink in their anal fin base indicating sexual maturity^12^.

Our initial playback (condition I) was a “natural” playback, combining the conspecific EOD waveform and conspecific SPI presented against a heterospecific EOD waveform and SPI. To then test if the EOD waveform alone is sufficient for species recognition, we tested focal fish in three different conditions (II–IV). In condition II, a conspecific EOD waveform and SPI was presented against a heterospecific EOD combined with a conspecific SPI. Condition III combined the conspecific EOD waveform with a heterospecific SPI presented against a heterospecific EOD waveform and heterospecific SPI. In condition IV, a con- and heterospecific EOD waveform were respectively combined with an artificial (scrambled) SPI to test if the EOD is both necessary and sufficient for species recognition. Finally, in condition V the heterospecific EOD was paired with a con- and heterospecific SPI to test if the SPI is both necessary and sufficient for species recognition (Fig. 4).

Behavioural experiments were conducted in an observation aquarium (L 160 × H 50 × W 50 cm; conductivity: 690 ± 20 μS/cm; temperature: 26 ± 1 °C) divided by plastic, grid partitions into three sections (Fig. 4; tank sections A, B–D, E). The playback electrodes were placed in the far right and left partitions (Fig. 4; tank sections A, E). The centre section (Fig. 4; tank sections B-D) was not physically divided, but ‘preference zones’ of 32 cm each were marked on the exterior of the tank. The focal fish was transferred to the tank 3.5 hours before each experiment began for acclimation. There was a clear plastic tube for shelter centred equidistant to the two playback electrodes.

For playback, we used a randomly selected pre-recorded active phase SPI (Spike2 and MICRO 1401) to trigger the outputs of a waveform generator (DG1032Z, Rigol Technologies, Beijing, China). The EOD waveforms were pre-recorded, averaged waveforms (across approximately 400 EODs) and could be triggered on two separate channels simultaneously. The signals were isolated from ground (2200 Analog Stimulus Isolator, A–M Systems, Carlsborg, WA, USA) and played into the water through dipole carbon electrodes spaced 104 cm apart, meaning the two electrodes were each 52 cm from the centre of the tank (Fig. 4; tank sections A, E). Each electrode was oriented with positive and negative poles (3.5 cm apart) parallel to the long axis of the aquarium. The output amplitude of each channel was adjusted to be level and match that of a live fish at equal distance (52 cm).

To control for side bias, the synthesized playback was presented on both sides of the tank (section A or E) in a randomized sequence equally often. All other possible discriminators were kept constant across all conditions. Each experimental playback began when the focal fish was in the shelter.

During preference experiments, *C. compressirostris* (n = 12) and *C. tamandua* (n = 10) males were presented with a 120-second playback sequence in 8 intervals, with a two minute pause between each interval for a total playback time of 16 minutes per condition. Males were video captured (Microsoft LifeCam HD-3000 webcam; Microsoft LifeCam software) under infrared illumination (880 nm). The total time each focal male spent in the pre-defined preference zones was analysed post-hoc using custom written MATLAB routines. The first condition began 30 minutes after night simulation. Between each of the five conditions was a 30 minute pause.

### Data analysis – Recordings of discharge behaviour

For each of the 29 resting phase SPIs, we used the time stamp occurrence of each EOD in a given sequence to calculate the inter-pulse-interval of the EODs (IPI) as:

1 $$IP{I}_{i}={t}_{i}-{t}_{i-1}$$

where *t* is the time at which an EOD occurred and *i* is the index of the given EOD. Sequences of IPIs were then plotted as a function of time and visually scanned for SPI patterns that have been linked to specific behavioural contexts in previous research. These included bursts (brief accelerations, scallops, rasps, etc.), cessations, pulse pairs, regularizations, and random SPI patterns^14,27^. IPI histograms (bin width 2 milliseconds) were also plotted, separately for species as well as sex. Population averages (arithmetic mean) and errors (standard error of the mean, SEM; standard deviation, STD) were calculated at a resolution of 2 milliseconds.

Principle Component Analyses (PCAs) are often used to identify communication signal differences between individuals and species, including in fish^65,66^, bird^67^, and insect^3,68^ species. We therefore investigated possible species and sex variation in the resting phase SPIs of our weakly electric fish using a PCA. Five variables were incorporated: (1) the total number of EODs produced in the 120-second sequence, (2) the mode inter-pulse-interval (i.e. the most frequent EOD interval), (3 & 4) the range (i.e. longest and shortest interval between two adjacent EODs), and (5) the number of runs (Table 1). For the runs analysis, IPIs were compared in sequential pairs; if the second interval in a pair was larger, smaller, or equal in duration to the first interval, it was assigned as *positive*, *negative*, or *equal*, respectively. A run was then defined as a series of increasing, decreasing, or constant IPIs. This definition of a run is derived from the runs test, an established statistical test to determine if two or more nominal events occur in random sequence or if the probability of a given event is a function of the outcome of a previous event^69,70^. Similar methods have been used previously to investigate the temporal organization of successive IPIs^36,71^. Two PCAs were conducted with these five variables clustered by species (n = 17 *C. compressirostris*; n = 12 *C. tamandua*) or by sex and species (n = 13 *C. compressirostris* males; n = 4 *C. compressirostris* females; n = 10 *C. tamandua* males; n = 2 *C. tamandua* females). Both PCAs were calculated using the *prcomp* function in R (singular value decomposition)^72^. All five SPI characteristics were log transformed to centre and scale variables. We calculated the Pearson correlation coefficient for each pair of variables using the R add-on package psych^73^. To visualize the data, we used the R add-on package ggbiplot^74^. A normal contour line with an ellipse probability of 68% for each defined group is visualized.

We additionally tested whether SPIs were the result of a renewal process or subject to memory based effects by calculating serial correlations (SC) from a given IPI sequence. Under the assumption of a renewal process, the EOD IPIs would be random; memory effects would result in dependencies of a given EOD IPI to its past. SC were calculated as a function of the distance between two IPIs (i.e., the lag) *i* according to:

2 $$S{C}_{i}=\frac{\langle IP{I}_{k}IP{I}_{k+i}\rangle -{\langle IP{I}_{k}\rangle }^{2}}{VAR(IPI)}$$

where *k* is the index of a given interval and 〈…〉 means the average over all *k*. Species-specific population averages for active and resting phase recordings were obtained as a function of lag *i*. To test whether the results differed significantly from chance level, we recalculated serial correlations according to the above formula, while randomly permuting the IPI sequences 250 times. From this, chance level was defined as the 99% confidence interval above the mean, averaged over all possible intervals (red lines in Fig. 3a–e).

We calculated the average duration of the SC for each recording by multiplying the interval number at which serial correlations first decreased under chance level with the median IPI interval duration in that specific recording. The distributions of SC durations for active and resting phase SPIs were compared between species with an individual non-parametric test (Kruskal-Wallis).

### Data analysis – Behaviour choice experiments

Across all five experimental playback conditions, the behaviour of the male focal fish was evaluated offline based on video recordings. We scored responses as total time spent within each pre-defined preference zone. Data was analysed according to Plath *et al*.^32^ using *strength of preference* (SOP) as a measure of association preference. SOP was calculated as:

3 $$SOP=\frac{{t}_{B}-{t}_{D}}{{t}_{B}+{t}_{D}}$$

where *t*_*B*_ is the time the focal fish spent in preference zone *B* and *t*_*D*_ is the time the focal fish spent in preference zone *D*. SOP values can range from −1 (complete avoidance of preference zone *B*) to 1 (complete preference of preference zone *B*). A one-sample *t*-test was used to compare SOP values to a random distribution of SOP = 0. Significant differences in the SOP values between conditions were tested with a repeated measures ANOVA, with individual male and SPI identifiers as random variables. If significant differences were identified, the post-hoc Tukey’s HSD pairwise comparison test was performed with a confidence interval of 95% using the R add-on package multcomp^75^. Data was tested for normality using the Shapiro-Wilk normality test and visualized with the *qqnorm* and *hist* functions in R^72^.

### Ethics statement

All experiments were approved by the Deputy for Animal Welfare of the University of Potsdam and are in accordance with the national legal requirements.

## Electronic supplementary material

Supplementary Information
