# Supplementary material for: Electric pulse characteristics can enable species recognition in African weakly electric fish species
Source: Sci Rep. 2018 Jul 17;8:10799. doi: 10.1038/s41598-018-29132-z (PMC6050243; doi:10.1038/s41598-018-29132-z)
Supplement: Supplementary file 1 — Supplementary Information [file 41598_2018_29132_MOESM1_ESM.doc]

Supplementary Information

Electric pulse characteristics can enable species recognition in African weakly electric fish species

Rebecca Nagel, Frank Kirschbaum, Volker Hofmann, Jacob Engelmann, Ralph Tiedemann

Table S1: 30-minute and 120-second SPI trains were recorded from two individuals on three separate dates to show individual variation in SPI statistics. comp = *C. compressirostris*; tam = *C. tamandua*; M = male.

| Individual ID, Sex | Date of Recording | 30 min or 120 sec analysis | Total Nr EODs | IPI (ms) | Range High (ms) | Range Low (ms) | Nr Runs |
| --- | --- | --- | --- | --- | --- | --- | --- |
|  |  |  | *total number of EODs recorded* | *predominant (mode) inter-EOD-interval recorded in the SPI* | *longest interval between two adjacent EODs* | *shortest interval between two adjacent EODs* | *total number of runs in the SPI; See main manuscript for more details* |
| comp64, M | 16.04.2016 | 120 s | 3346 | 13.854 ms | 171.230 ms | 10.036 ms | 2033 |
| comp64, M | 25.05.2016 | 120 s | 3804 | 13.406 ms | 189.528 ms | 10.352 ms | 2468 |
| comp64, M | 17.06.2016 | 120 s | 3504 | 11.316 ms | 192.754 ms | 10.002 ms | 2153 |
| SD (Relative Range) |  |  | ± 232 (12.9%) | ± 1.4 (19.7%) | ± 11.6 (11.7%) | ± 0.2 (3.5%) | ± 224.0 (19.6%) |
|  | | | | | | | |
| comp64, M | 16.04.2016 | 30 min | 47205 | 12.516 ms | 1688.834 ms | 9.990 ms | 28976 |
| comp64, M | 25.05.2016 | 30 min | 50534 | 18.014 ms | 1985.430 ms | 9.996 ms | 32891 |
| comp64, M | 17.06.2016 | 30 min | 53332 | 11.344 ms | 2257.058 ms | 9.990 ms | 33202 |
| SD (Relative Range) |  |  | ± 3067 (12.2%) | ± 3.6 (47.8%) | ± 284.2 (28.7%) | ± 0.003 (0.06%) | ± 2355.2 (13.3%) |
|  | | | | | | | |
| tam08, M | 14.04.2016 | 120 s | 1236 | 135.336 | 449.420 | 11.198 | 870 |
| tam08, M | 09.06.2016 | 120 s | 1190 | 156.270 | 570.738 | 24.272 | 708 |
| tam08, M | 28.06.2016 | 120 s | 1298 | 149.170 | 412.540 | 10.124 | 795 |
| SD (Relative Range) |  |  | ±54 (8.7%) | ± 10.0 (14.2%) | ± 82.0 (33.1%) | ± 7.0 (93.1%) | ± 81.1 (20.5%) |
|  | | | | | | | |
| tam08, M | 14.04.2016 | 30 min | 19311 | 34.766 | 885.788 | 10.038 | 12755 |
| tam08, M | 09.06.2016 | 30 min | 17690 | 41.876 | 2997.284 | 12.856 | 10526 |
| tam08, M | 28.06.2016 | 30 min | 20283 | 38.004 | 2576.498 | 10.010 | 12257 |
| SD (Relative Range) |  |  | ± 1309 (13.6%) | ± 3.5 (18.6%) | ± 1117.5 (98.1%) | ± 1.6 (25.9%) | ± 1169.9 (18.8%) |

Table S2: Correlation matrix (above) and p-values (below) for the five variables included in our principle component analysis. The p-values above the diagonal are adjusted for multiple tests using the Holm correction.

| Correlation matrix | Total number of EODs | Frequency Peak | Range (high) | Range (low) | Number of Runs |
| --- | --- | --- | --- | --- | --- |
| Total number of EODs | 1.00 |  |  |  |  |
| Frequency Peak | -0.86 | 1.00 |  |  |  |
| Range (high) | -0.15 | 0.04 | 1.00 |  |  |
| Range (low) | -0.78 | 0.80 | 0.05 | 1.00 |  |
| Number of Runs | 0.99 | -0.84 | -0.10 | -0.75 | 1.00 |

| P-values | Total number of EODs | Frequency Peak | Range (high) | Range (low) | Number of Runs |
| --- | --- | --- | --- | --- | --- |
| Total number of EODs | - | *p* < 0.0001 | 1.00 | *p* < 0.0001 | *p* < 0.0001 |
| Frequency Peak | *p* < 0.0001 | - | 1.00 | *p* < 0.0001 | *p* < 0.0001 |
| Range (high) | 0.4373 | 0.8218 | - | 1.00 | 1.00 |
| Range (low) | *p* < 0.0001 | *p* < 0.0001 | 0.7779 | - | *p* < 0.0001 |
| Number of Runs | *p* < 0.0001 | *p* < 0.0001 | 0.5962 | *p* < 0.0001 | - |

**comp01** c


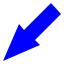


|  | 200. |
| --- | --- |
|  | 150. |
| Interval (s) | 100. |
|  | 050. |
|  | 000. |

|  |  |  |  |  |  |  |  |  |  |  |  |  |  |  |  |
| --- | --- | --- | --- | --- | --- | --- | --- | --- | --- | --- | --- | --- | --- | --- | --- |
|  |  |  |  |  |  |  |  |  |  |  |  |  |  |  |  |
|  |  |  |  |  |  |  |  |  |  |  |  |  |  |  |  |
|  |  |  |  |  |  |  |  |  |  |  |  |  |  |  |  |
|  |  |  |  |  |  |  |  |  |  |  |  |  |  |  |  |
|  |  |  |  |  |  |  |  |  |  |  |  |  |  |  |  |
|  |  |  | r |  |  |  |  |  |  |  |  |  |  |  |  |
|  | 0 | |  | 20 | | 40 ^b^ | | 60 | | 80 | | 100 | | 120 | |
|  |  |  |  |  |  |  |  | Time (s) | |  |  |  |  |  |  |


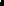

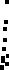

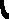

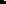

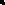

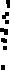

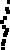

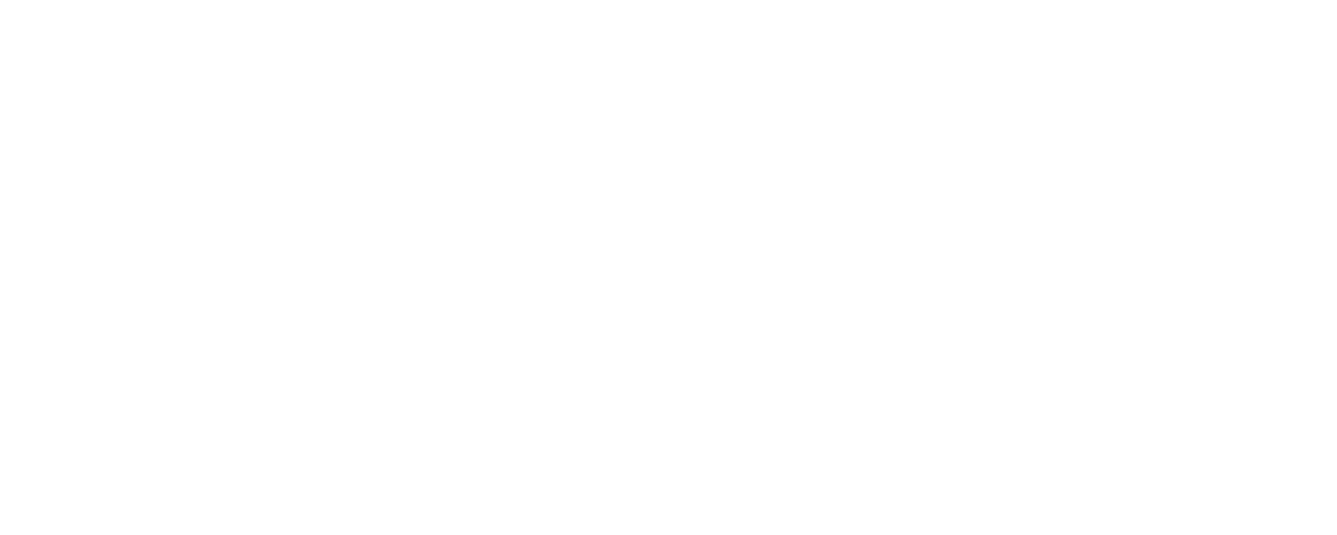

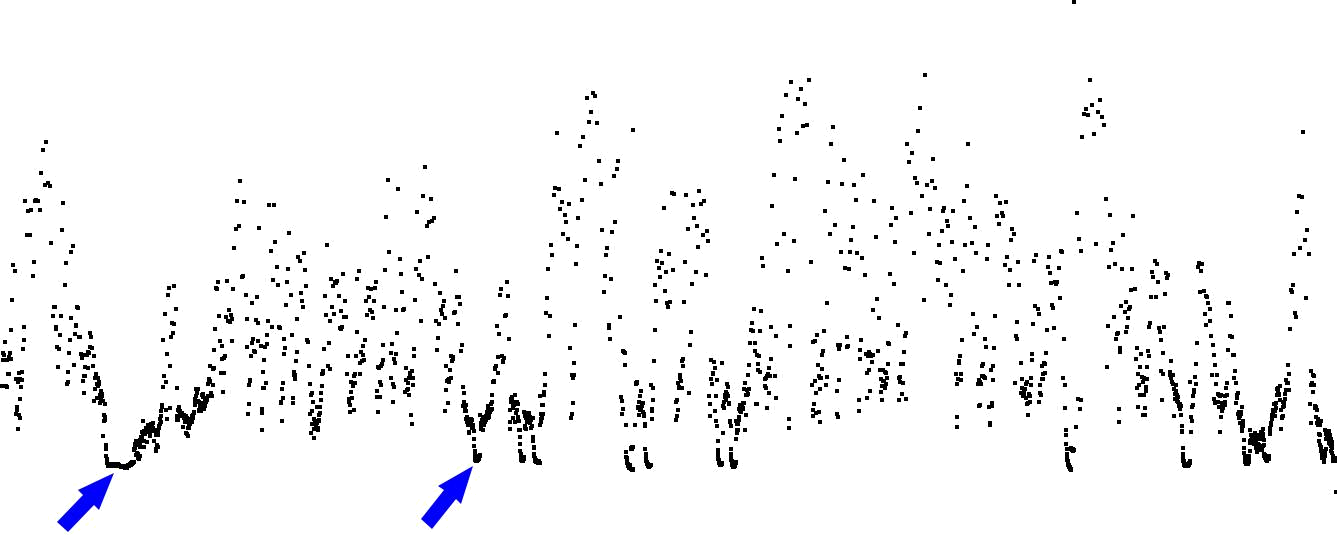


**comp03**

**


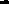

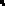
**

|  | 150. |
| --- | --- |
| Interval (s) | 100. |
|  | 050. |
|  | 000. |


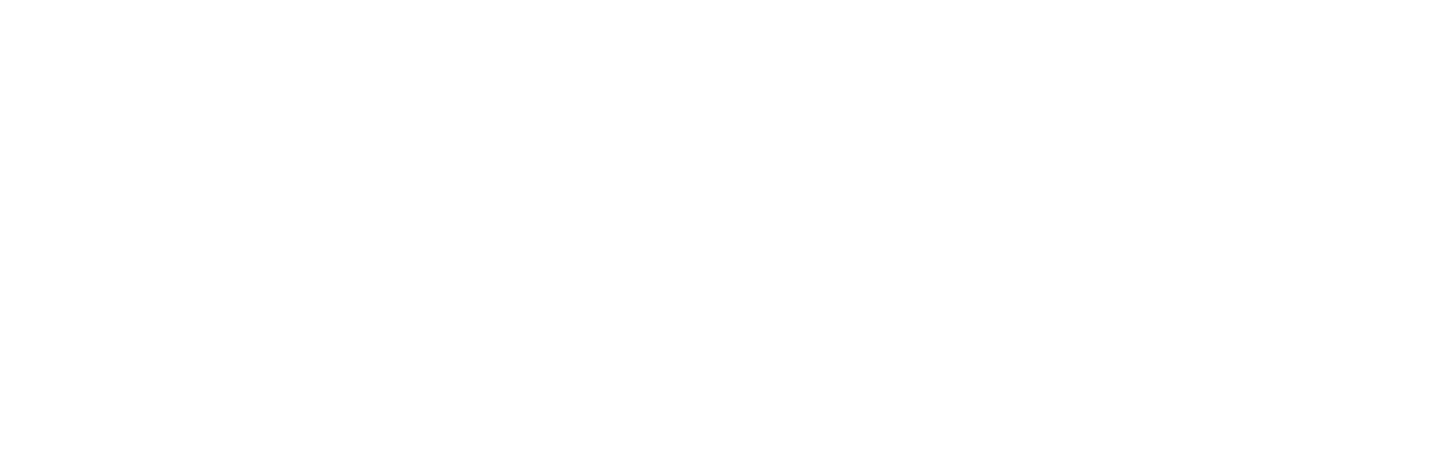

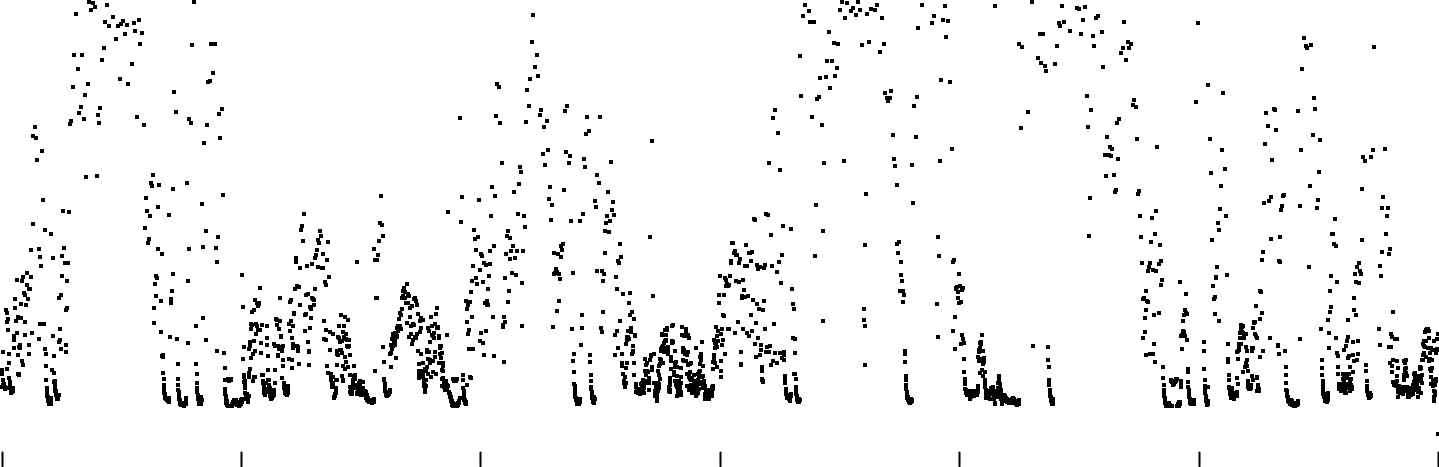


0 20 40 60 80 100 120

Time (s)

**comp04**

**


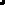


**

|  | 150. |
| --- | --- |
| Interval (s) | 100. |
|  | 050. |
|  | 000. |


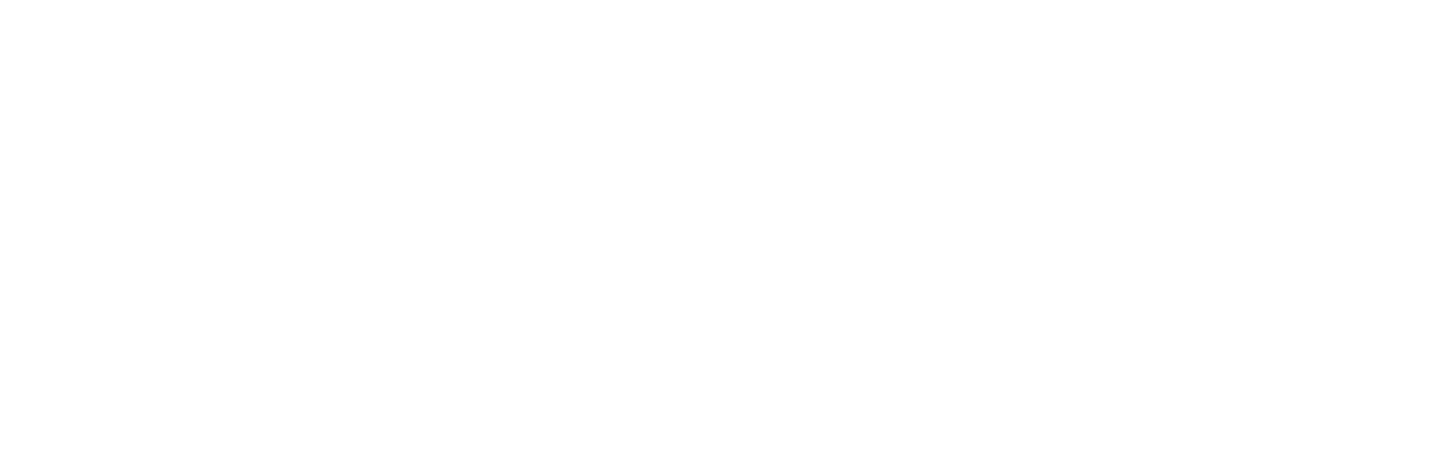

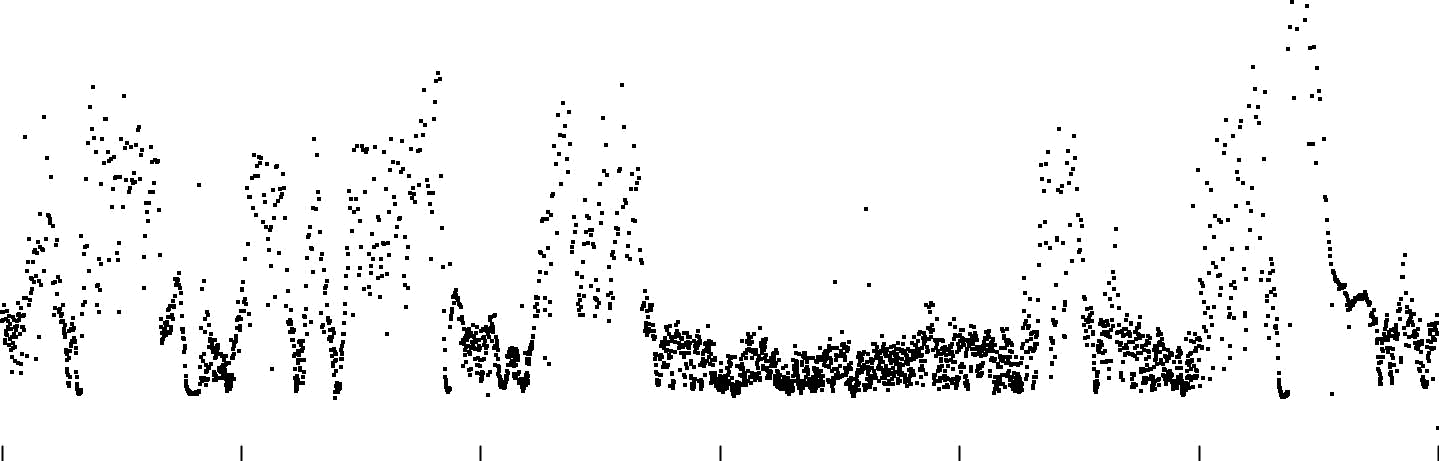


0 20 40 60 80 100 120

Time (s)

**tam04**

**

**

|  | 40. |
| --- | --- |
|  | 30. |
| Interval (s) | 20. |
|  | 10. |
|  | 00. |


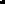


0 20 40 60 80 100 120

Time (s)

**tam05**

|  | 40. |
| --- | --- |
|  | 30. |
| Interval (s) | 20. |
|  | 10. |
|  | 00. |

0 20 40 60 80 100 120

Time (s)

**tam07**

|  | 40. |
| --- | --- |
|  | 30. |
| Interval (s) | 20. |
|  | 10. |
|  | 00. |

0 20 40 60 80 100 120

Time (s)

Figure S1: Example sequences of inter-pulse-intervals recorded during the day (i.e. during the resting period) for *C. compressirostris* (comp) and *C. tamandua* (tam) individuals. The title of each plot is the individual’s ID number. Each point is one interval. An example of bursts (b), cessations (c), and regularized (r) inter-pulse-intervals are denoted in the first panel.

Figure S2: Inter-EOD-interval histograms from resting phase SPIs. Panels show relative histograms (bin width 2 milliseconds) of the inter-EOD-interval for individual *C. tamandua* males.
